# Icariin and Icariside II Reciprocally Stimulate Osteogenesis and Inhibit Adipogenesis of Multipotential Stromal Cells through ERK Signaling

**DOI:** 10.1155/2021/8069930

**Published:** 2021-12-16

**Authors:** Dawei Zhang, Ning Zhao, Chao Wan, Jikun Du, Jiantao Lin, Hongbo Wang

**Affiliations:** ^1^Shenzhen Hospital of Integrated Traditional Chinese and Western Medicine, Guangzhou University of Chinese Medicine, Shenzhen 518104, China; ^2^Guangdong Key Laboratory for Research and Development of Natural Drugs, The Marine Biomedical Research Institute, Guangdong Medical University, Zhanjiang 524023, China

## Abstract

Herba Epimedii is a famous Chinese herbal medicine for treating bone diseases. Icariin and icariside II, the main chemical constituents, have attracted great attention from scientists for their potential as antiosteoporosis agents. Our study aimed to evaluate their effects on the lineage commitment of multipotential stromal cells (MSCs). The osteogenesis and adipogenesis of MSCs were assessed by ALP activity, calcium deposition, and adipocyte formation. The expression profiles and levels of osteogenic and adipogenic specific genes were evaluated by cDNA microarray and quantitative real-time PCR. The involvement of extracellular signal-regulated kinase (ERK) signaling was studied by enzyme-linked immunosorbent assay. Icariin and icariside II significantly increased ALP activity and mineralization during osteogenic differentiation of MSCs. *Runx2*, *Col1*, and *Bmp2* were upregulated in the presence of icariin and icariside II. Meanwhile, they downregulated *Pparg*, *Adipsin*, and *Cebpb* expression during adipogenic differentiation. cDNA microarray revealed 57 differentially expressed genes during lineage commitment of MSCs. In addition, icariin and icariside II enhanced the phosphorylation of ERK, and the above biological effects were blocked by ERK inhibitor U0126. Icariin and icariside II may drive the final lineage commitment of MSCs towards osteogenesis and inhibit adipogenesis through the ERK signaling pathway. Both of them exert multiple osteoprotective effects and deserve more attention for their medicinal and healthcare prospects.

## 1. Introduction

The bone is a highly dynamic tissue which undergoes constant remodeling all through life. Normal bone remodeling requires the appropriate balance between bone formation and resorption [1]. Impairment in the bone remodeling process often results in abnormal metabolic bone diseases, especially osteoporosis. Osteoporosis, particularly for postmenopausal women, accelerates the bone resorption process and increases the risk of bone fragility and fractures. Therapeutic options for osteoporosis include antiresorptive agents (bisphosphonates, raloxifene, and estrogen) and anabolic agents (teriparatide and abaloparatide) [2]. However, the pharmaceutics are associated with various side effects and high costs [3].

Herba Epimedii (Yin Yang Huo) has been prescribed for impotence, arthritis, numbness, and weakness of the limbs for centuries in China. According to Chinese Pharmacopoeia, Herba Epimedii is derived from the aerial part of the *Epimedium* species including *E. koreanum* Nakai, *E. brevicornum* Maxim, and *E. sagittatim* (Sieb & Zucc.) Maxim. As the most frequently used Chinese medicinal herb for osteoporosis and fracture, 85 clinical trials were conducted using traditional Chinese medicine prescriptions containing Herba Epimedii to treat primary and secondary osteoporosis from 2005 to 2016 [4]. Many studies have been performed to investigate the antiosteoporosis effects of Herba Epimedii extracts and chemical constituents, especially icariin, in various animal and cell models. Herba Epimedii aqueous extract (110 mg/kg/d) protected bone loss in ovariectomized (OVX) rats by improving trabecular microarchitecture and urinary calcium excretion [5]. In addition, total flavonoids of Herba Epimedii (50 or 100 *μ*g/g) were able to suppress urinary calcium excretion as well as loss of bone mass and strength at the distal femur and improve trabecular bone microarchitecture in OVX mice [6]. Administration of icariin (125 mg/kg/d) significantly increased biomechanical strength and improved biochemical and histopathological parameters in OVX rats [7]. Chen et al. [8] also reported that icariin (125, 250, and 500 mg/kg/d) significantly increased bone mineral density, biomechanical strength, and relieved trabecular microarchitecture deterioration in OVX rats.

Many studies have revealed that flavonoid components of Herba Epimedii exhibited osteoprotective effects by stimulating osteoblast differentiation and suppressing osteoclast differentiation [9–11]. In recent years, there has been an increased amount of interest in the multipotent differentiation capabilities of multipotential stromal cells (MSCs). Flavonoids of Herba Epimedii were reported to stimulate the osteogenesis of human MSCs [12]. Icariin and rat MSCs combined treatment promoted angiogenesis and neurogenesis after cerebral ischemia by reducing brain infarction volume and improving neurologic deficits of motor and somatosensory function [13]. Icariin was able to protect against iron overload induced apoptosis and dysfunction of MSCs [14]. Icariin (10 *µ*M) may recover the decreased osteogenic differentiation and bone formation function of MSCs from OVX rats [15]. However, these studies were focused on the protective and osteogenic effects of icariin in MSCs. The reciprocal regulation potential of icariin and its metabolites during osteogenesis and adipogenesis of MSCs remains unclear. Previously, icariin and its metabolites were investigated [16] and reported to regulate the lipid metabolism [17]. Notably, we found that icariin significantly suppressed adipocytic transdifferentiation of osteoblasts [18], which suggests that icariin may have the potential to regulate adipogenesis. Studies have demonstrated that there may exist a reciprocal relationship between osteogenesis and adipogenesis of MSCs [19, 20], and the extracellular signal-regulated kinase (ERK) signaling is closely related to this process [21]. Herein, we aimed to investigate how icariin and its main metabolite (icariside II) modulate the lineage commitment of MSCs.

## 2. Materials and Methods

### 2.1. Cell Culture

MSCs were isolated from 6-week-old Kunming mice as previously described [22, 23]. Animal care and the procedure were approved by the Institutional Animal Care and Use Committee (December 1^st^, 2019). Briefly, mice were euthanized, and both femora and tibiae were aseptically harvested. Bone marrow cells were flushed out with Dulbecco's modified Eagle medium (Gibco, Scotland, UK) supplemented with 10% heat-inactivated and charcoal-stripped fetal bovine serum (Gibco), 1% penicillin-streptomycin (culture medium). MSCs were identified by flow cytometry and differentiation capability and maintained in the culture medium at 37°C. Sodium fluoride (NaF) and 17*β*-estradiol (ES) served as positive control for different experiments, and PBS was used as vehicle control [22].

### 2.2. Proliferation Assay

MSCs were seeded in 96-well plates (1 × 10^6^ cells/well) and then incubated for 48 h with 0.01, 0.1, 1, and 10 *µ*M icariin or icariside II (purity >97%, previously isolated from *Epimedium koreanum* Nakai [24]), respectively. Then, 3-[4, 5-dimethylthiazol-2-yl]-2,5 diphenyl tetrazolium bromide (MTT, 5.0 mg/mL, Sigma-Aldrich, St. Louis, MO, USA) was added and further incubated for 4 h. Finally, the medium was replaced with dimethyl sulfoxide. Absorbance was measured at 570 nm.

### 2.3. Osteogenic Differentiation

Cells were seeded in 48-well plates (2.5 × 10^6^ cells/well). After reaching 80% confluence, the medium was changed to the fresh medium with osteogenic supplements (OS: 5 mM *β*-glycerophosphate, 0.1 *µ*M dexamethasone, and 50 mg/mL ascorbic acid, Sigma-Aldrich), ES (10 nM), NaF (1 *µ*M), and icariin or icariside II (0.01–10 *µ*M). Cultures were maintained for 7 d. Cells were washed with PBS and then lysed by two freeze-thaw cycles. ALP activity and protein content were determined using a commercial ALP kit (Nanjing Jiancheng Bioengineering Institute, Nanjing, China) and by the bicinchoninic acid method (Beyotime Biotechnology, Shanghai, China), respectively, and ALP activity was normalized for total protein concentration [18].

### 2.4. Alizarin Red S Staining

MSCs were seeded as described in 2.3 and cultured for 21 d in the OS medium. The presence of mineralized nodules was evaluated by alizarin red S (ARS, Sigma-Aldrich) staining [18]. After being fixed in 70% ethanol for 30 min, cells were washed with PBS and stained with ARS (40 mM, pH 4.2) for 30 min. Images were acquired using the Eclipse Ti microscope (Nikon, Tokyo, Japan) connected to a video camera. The ARS staining was eluted with 10% (w/v) cetylpyridium chloride (Sigma-Aldrich) for quantitative analysis. The absorbance was measured at 570 nm.

### 2.5. Adipogenic Differentiation

Cells were seeded in 48-well plates (3 × 10^6^ cells/well) and cultured for 14 d in the medium containing adipogenic supplements (AS: 0.1 *µ*M dexamethasone and 10 mg/L insulin, Sigma-Aldrich), ES (10 nM), NaF (1 *µ*M), and icariin or icariside II (0.01–10 *µ*M). Fat droplets were visualized by oil red O staining [22]. After being fixed with 10% formalin for 30 min, cells were washed PBS and stained with oil red O (0.6% (w/v) in 60% isopropanol) for 15 min. After being eluted with 100% (v/v) isopropanol, the absorbance was measured at 510 nm.

### 2.6. Quantitative Real-Time PCR (qPCR) Analysis

Total RNA was obtained from MSCs using TRIzol reagent (Invitrogen, Carlsbad, CA, USA) and reverse transcripted into cDNA using PrimeScript^TM^ RT reagent kit (TaKaRa, Dalian, China). qPCR assay was carried out using SYBR^®^ Premix Ex Taq^TM^ II kit (TaKaRa). Primer sequences were according to a previous publication [23]. Relative quantification of mRNA expression was calculated as the fold change using the 2^−ΔΔ*Ct*^ method [25].

### 2.7. Enzyme-Linked Immunosorbent Assay (ELISA) Analysis

Levels of phospho-ERK 1/2 and total ERK 1/2 proteins in cell lysis were determined using ERK1/2 (pT202/Y204 + total) ELISA kit (Abcam, Cambridge, UK). Phosphorylated ERK was normalized to total ERK protein and normalized by the control group.

### 2.8. cDNA Microarray Analysis

MSCs were incubated for 3 d with icariin (1 *µ*M) in the AS or OS medium, respectively. Total RNA was extracted and reverse-transcripted to cDNA with SuperScript III reverse transcriptase (Invitrogen). Cy3-dUTP or Cy5-dUTP (Amersham Pharmacia Biotech, Piscataway, NJ) was incorporated during reverse transcription of 40 *μ*g of purified total RNA. Different fluorescent-labeled cDNA samples were mixed and competitively hybridized to a homemade cDNA microarray [26], which contained 1000 genes selected from a mouse clone set. Microarrays were scanned using a microarray scanner (ScanArray 4000, GSI Lumonics, MA), and images were analyzed with GenePix Pro 4.0 software (Axon, CA). Genes showing greater than 2-fold induction or repression (Cy5/Cy3 ratios > 2 or <0.5) were selected for further analysis. The Protein Analysis Through Evolutionary Relationships (PANTHER) classification system was applied to analyze the functional classification and correlated pathways (http://www.pantherdb.org) [27].

### 2.9. Statistical Analysis

Experiments were performed in three replicates, and all data were expressed as mean ± standard deviation (SD). The differences between multiple groups were assessed using one-way analysis of variance (ANOVA) or Student's *t*-test. A level of *p* < 0.05 was classified as statistically significant.

## 3. Results and Discussion

To evaluate how icariin and icariside II regulate the lineage commitment of MSCs, primary MSCs were isolated, subjected to proliferation assay, and induced to differentiate into adipogenic and osteogenic lineages.

### 3.1. Icariin and Icariside II Stimulated the Proliferation of MSCs

**Figure 1:**
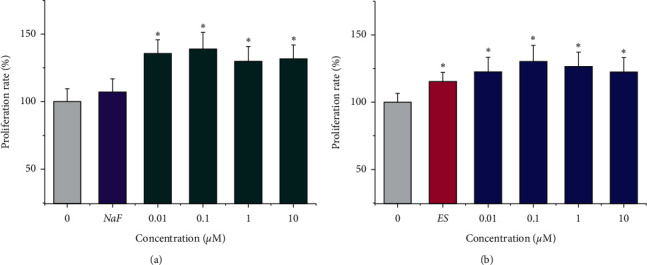
Icariin (a) and icariside II (b) stimulated the proliferation of MSCs. NaF, 1 *µ*M; ES, 10 nM 17*β*-estradiol. ^*∗*^*P* < 0.05 vs. control.

Cells were incubated with icariin, icariside II, 17*β*-estradiol (ES), and NaF for 2 d. As shown in Figure 1, 10 nM ES increased the proliferation rate of MSCs to 15.2% versus PBS control (*p* < 0.05). In contrast, 0.01–10 *µ*M icariin and icariside II more potently stimulated the proliferation of MSCs up to 30% (*p* < 0.05), respectively. The maximum effect was obtained at 0.1 *µ*M for both icariin and icariside II, showing a tendency to decrease with increasing concentrations. Wei et al. [28] reported that icariin (0.1 *µ*M) effectively induced the proliferation of rat MSCs from 1 to 14 days (*p* < 0.05). Studies also indicated that increasing icariin concentrations, for instance, over 20 *µ*M may exhibit a negative effect on rat MSCs cell growth [29], and 0.001 *µ*M had no significant effect [30]. Therefore, the optimal concentrations for cell proliferation may be 0.01–10 *µ*M.

### 3.2. Icariin and Icariside II Promoted the Osteogenic Differentiation of MSCs

**Figure 2:**
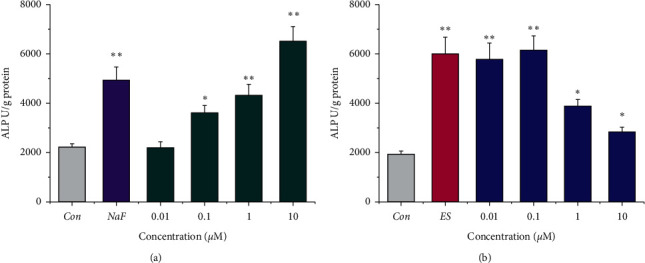
Icariin (a) and icariside II (b) increased ALP activity during osteogenesis of MSCs. NaF, 1 *µ*M; ES, 10 nM 17*β*-estradiol. ^*∗*^*P* < 0.05 and ^*∗∗*^*p* < 0.01 vs. PBS control.

Previously, our group [22] and Zhang et al. [10] found that total flavonoids of Herba Epimedii significantly stimulated the osteogenic differentiation of mouse and human MSCs, respectively, among which icariin and icariside II were the main constituents. Another study indicated that 1 *µ*M icariin may induce osteogenesis of rat MSCs by increasing ALP activity and calcium deposition [30]; however, no other concentrations and positive control were evaluated. Osteogenesis of MSCs was first quantified by monitoring ALP activity, an early marker. As shown in Figure 2, both ES and NaF potently increased ALP activity to 3.1 and 2.2-fold, respectively (*p* < 0.01). Icariin (0.1–10 *µ*M) stimulated ALP of MSCs in a concentration-dependent manner. Icariside II (0.01–10 *µ*M) also significantly induced the ALP activity of MSCs, among which 0.1 *µ*M was the most potent.

**Figure 3:**
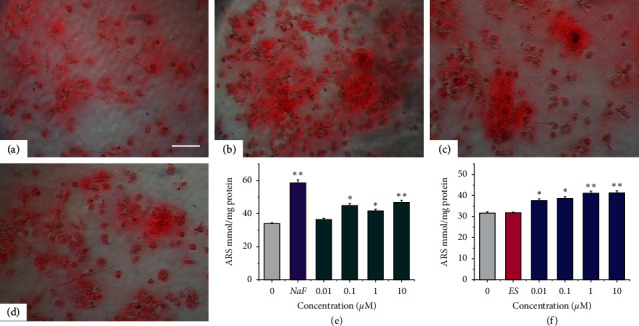
Quantification of alizarin red S staining during osteogenesis of MSCs. (a–d). Alizarin red S staining (100×): (a) Osteogenic supplements (OS) medium. (b) OS + NaF (1 *µ*M). (c) OS + icariin (1 *µ*M). (d) OS + icariside II (1 *µ*M). (e-f) Quantification of ARS staining: (e) Icariin. (f) Icariside II. ^*∗*^*P* < 0.05 and ^*∗∗*^*p* < 0.01 vs. the OS group. Scale bar = 20 *µ*M.

Osteogenic differentiation of MSCs was further characterized by ARS staining to detect extracellular matrix (ECM) mineralization (Figure 3). Osteogenic supplements intensively increased calcium deposition as indicated by ARS staining (Figure 3(a)). In addition, a robust number of mineralized nodules were found in NaF, icariin, and icariside II groups (Figures 3(b)–3(d)).

Quantification of ARS staining confirmed that 1 *µ*M NaF potently increased the calcium deposition in ECM during osteogenic differentiation of MSCs (Figure 3(e)). Similarly, 0.1–10 *µ*M icariin significantly promoted the calcium deposition in ECM (Figure 3(e), *p* < 0.05), which was confirmed by another study that 0.01–1 *µ*M icariin stimulated the calcium deposition in rat MSCs [28]. In addition, calcium deposition in ECM was also significantly induced by 0.01–10 *µ*M icariside II in a concentration-dependent manner (Figure 3(f)). At the concentration of 10 *µ*M, both icariin and icariside II reached the maximum effect, increasing the calcium content by 37.9% and 30.2%, respectively (*p* < 0.01). Taken together, both 0.1–10 *µ*M icariin and 0.01–10 *µ*M icariside II may significantly promote osteogenesis of MSCs by enhancing early marker-ALP activity and final marker-ECM mineralization.

### 3.3. Icariin and Icariside II Suppressed the Adipogenic Differentiation of MSCs

Although Herba Epimedii has long been used in traditional Chinese medicine for the bone and joints, recent data have shown that Herba Epimedii may offer potential benefits for obesity. Herba Epimedii extract (100, 200 *µ*g/mL) and icariin (50, 100 *µ*M) were reported to inhibit the adipocyte differentiation of 3T3-L1 preadipocytes by decreasing the expression of the adipogenic transcription factors [31]. One study found that icariin was a novel peroxisome proliferator-activated receptor-alpha (PPARa) agonist which activated lipid metabolism gene expressions in mice [32]. Another study reported that icariin induced irisin/FNDC5 expression in C2C12 cells, indicating that icariin may protect against obesity [33]. Previously, our group found that total flavonoids of Herba Epimedii significantly suppressed the adipogenic differentiation of mouse MSCs [22], and icariin may suppress adipocytic transdifferentiation of primary osteoblasts [18]. Therefore, available data suggest that icariin may play an essential role in adipogenesis.

**Figure 4:**
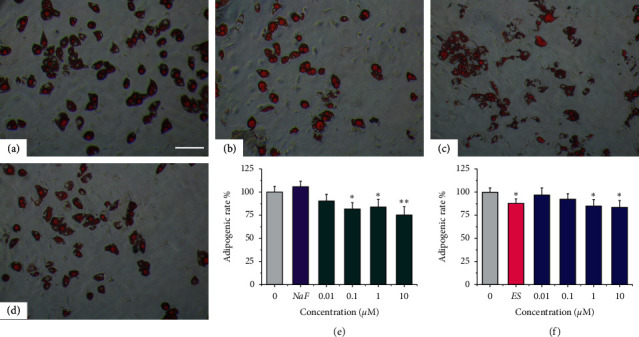
Adipocyte-like cell formation during adipogenesis of MSCs. (a–d) Oil red O staining (100×). (a) AS medium. (b) AS + ES (10 nM). (c) AS + icariin (1 *µ*M). (d) AS + icariside II (1 *µ*M). (e-f) Quantification of oil red O staining: (e) Icariin. (f) Icariside II. ^*∗*^*P* < 0.05 and ^*∗∗*^*p* < 0.01 vs. the AS group. Scale bar = 40 *µ*M.

MSCs were capable of adipogenic differentiation in the adipogenic supplements (AS) medium characterized by oil red O staining. In Figures 4(a)–4(d), plenty of reddish-brown droplets were formed and accumulated in the AS group, which were decreased by the addition of test samples. Quantitative analysis revealed that 0.1–10 *µ*M icariin and 1–10 *µ*M icariside II significantly suppressed the adipogenic differentiation of MSCs (Figures 4(e)–4(f), *p* < 0.05), which was slightly increased with concentration. Notably, the adipogenic inhibitory effect of experimental groups was as follows: icariin (10 *µ*M) > icariside II (10 *µ*M) > ES (10 nM).

### 3.4. Gene Expression

The differentiation balance of MSCs, especially to osteogenic and adipogenic lineages, is crucial to bone hematopoiesis. Runt-related transcription factor 2 (*Runx2*) and *Pparg* are generally regarded as the key regulators of osteogenesis and adipogenesis, respectively. Studies have demonstrated an inverse relationship between *Runx2* and *Pparg* expression during osteogenesis and adipogenesis of MSCs [19, 34]. Our previous studies showed that flavonoids of Herba Epimedii promoted the osteogenesis of MSCs by increasing the *Runx2* and bone morphogenetic protein 2 (*Bmp2*) gene expression while downregulating the expression of *Pparg* [22].

**Figure 5:**
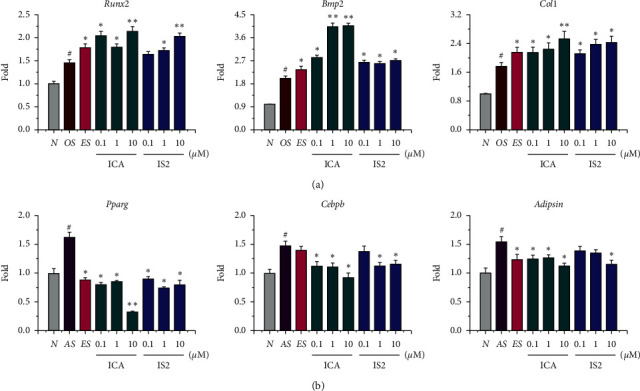
Representative gene expression during osteogenesis (a) and adipogenesis (b) of MSCs in the presence of icariin and icariside II. AS, adipogenic supplement; OS, osteogenic supplement; ES, 10 nM 17*β*-estradiol; N, growth medium; ICA, icariin; IS2, icariside II. ^#^*P* < 0.01 vs. the N group. ^*∗*^*P* < 0.05 and ^*∗∗*^*p* < 0.01 vs. the AS/OS group.

As shown in Figure 5, qPCR assay indicated that icariin and icariside II induced the osteogenic and adipogenic bidirectional differentiation of MSCs through modulating osteogenic and adipogenic-related gene expressions. The addition of ES, icariin, and icariside II significantly upregulated the expression of *Runx2*, collagen type 1 (*Col1*), and *Bmp2* (Figure 5(a)). It is noticeable that 10 *µ*M icariin upregulated *Runx2*, *Col1*, and *Bmp2* expression to 2.1, 2.5, and 4.1-fold, respectively. No significant difference between icariin and icariside II was found.

Similarly, representative adipogenic marker genes, *Pparg*, CCAAT/enhancer-binding protein beta (*Cebpb*), and *Adipsin*, were markedly upregulated in the AS medium (Figure 5(b)). In comparison, ES treatment decreased the expression of *Pparg* and *Adipsin*. Both icariin and icariside II downregulated the expression of *Pparg*, *Cebpb*, and *Adipsin*. For instance, icariin (10 *µ*M) and icariside II (1 *µ*M) decreased the expression of *Pparg* by 67.9% and 26.2% versus the control group, respectively.

### 3.5. ERK Signaling Was Involved during MSCs Osteogenic and Adipogenic Differentiation

The ERK signaling pathway is critically involved in the commitment of MSCs into the osteogenic lineage [35]. ERK signaling could enhance ALP activity in osteoblast progenitor cells and MSCs by activating the osteogenic-related transcription regulators [36, 37]. Jaiswal et al. [21] found that inactivation of ERK blocked the osteogenic differentiation but induced the adipogenesis of adult human MSCs.

**Figure 6:**
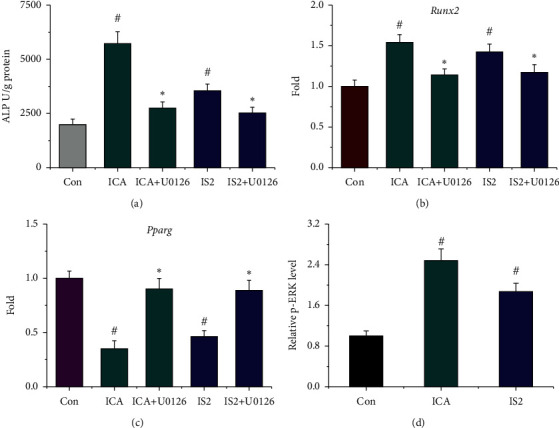
ERK signaling was activated during osteogenesis and adipogenesis of MSCs in the presence of icariin and icariside II. (a) ALP activity. (b-c) Gene expression of *Runx2* and *Pparg*. (d) Level of ERK phosphorylation by ELISA. ICA, icariin; IS2, icariside II; U0126, ERK1/2 inhibitor. ^*#*^*P* < 0.01 vs. the control group. ^*∗*^*P* < 0.05 vs. the ICA/IS2 group.

In Figures 6(a)–6(c), pretreatment with 10 *μ*M U0126, specific ERK1/2 inhibitor, decreased the above biological effects of icariin and icariside II on ALP activity and gene expression of *Runx2* and *Pparg* (*P* < 0.05). In addition, icariin and icariside II treatment activated ERK signaling by increasing the phosphorylation of ERK (Figure 6(d), *P* < 0.01), which suggests that icariin and icariside II may drive the osteogenesis and adipogenesis of MSCs through ERK signaling.

### 3.6. Gene Expression Profiling during MSCs Osteogenic and Adipogenic Differentiation

**Figure 7:**
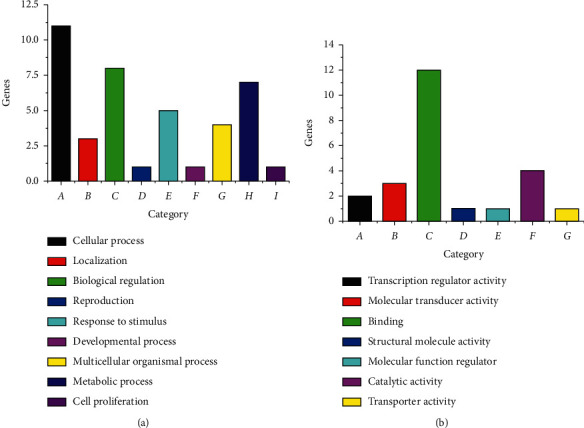
PANTHER functional analysis during osteogenic differentiation of MSCs. (a) Biological process. (b) Molecular function.

**Figure 8:**
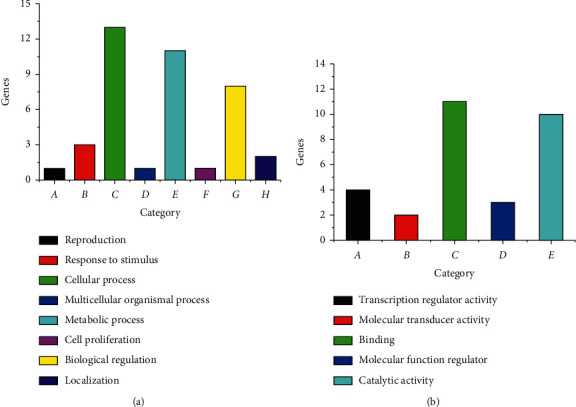
PANTHER functional analysis during adipogenic differentiation of MSCs. (a) Biological process. (b) Molecular function.

According to PANTHER analysis, 27 differentially expressed genes during MSCs osteogenesis were categorized into 41 biological processes and 24 molecular functions (Figure 7, Supplementary Materials Table 1). More specifically, important biological processes were the cellular process (26.8%), biological regulation (19.5%), metabolic process (17.1%), and response to stimulus (12.2%). In addition, binding accounted for as much as 50% of molecular functions. Similarly, 30 differentially regulated genes during MSCs adipogenesis were involved in 40 biological processes and 30 molecular functions (Figure 8, Supplementary Materials Table 2). Most of the above genes were related to the cellular process (32.5%), metabolic process (27.5%), and biological regulation (20.0%) in biological process and binding (36.7%) and catalytic activity (33.3%) in molecular function (Figure 8). The effects of icariin and icariside II on the above genes were under investigation. For example, during osteogenic differentiation of MSCs, icariin upregulated *Srebf1* and *Psma6* to 2.20 and 3.07-fold, respectively, while during adipogenic differentiation of MSCs, icariin downregulated *Marcks* and *Lpl* to 0.49 and 0.36-fold, respectively.

## 4. Conclusion

MSCs are self-renewing, multipotent precursors to various cell lineages, which have shown great promise in the field of tissue engineering and regenerative medicine. An imbalance between osteogenesis and adipogenesis of MSCs may lead to various metabolic diseases. Our results revealed the reciprocal regulation of osteogenesis and adipogenesis of MSCs by icariin and its main metabolite icariside II through ERK signaling. The gene expression profiles indicated that 57 genes were involved in the lineage commitment of MSCs. Previous studies strongly showed that Herba Epimedii flavonoids might restore bone homeostasis by promoting bone formation and suppressing bone resorption. These findings, taken together, contribute to a comprehensive evaluation of the osteoprotective effects of Herba Epimedii flavonoids. In summary, Herba Epimedii, especially its flavonoid components, has broad prospects of application in osteoporosis prevention and healthcare.

## Data Availability

The data used to support the findings of this study are available from the corresponding author upon request.

## Supplementary Materials

Supplementary Materials Figure 1. Chemical structure of icariin and icariside II. Table 1. Gene expression profiles during osteogenic differentiation of MSCs by cDNA microarray. Table 2. Gene expression profiles during adipogenic differentiation of MSCs by cDNA microarray. Supplementary material related to this article can be found in the online version.
