# Supplementary material for: Icariin and Icariside II Reciprocally Stimulate Osteogenesis and Inhibit Adipogenesis of Multipotential Stromal Cells through ERK Signaling
Source: Evid Based Complement Alternat Med. 2021 Dec 16;2021:8069930. doi: 10.1155/2021/8069930 (PMC8702327; doi:10.1155/2021/8069930)
Supplement: Supplementary Materials — Figure 1. Chemical structure of icariin and icariside II. Table 1. Gene expression profiles during osteogenic differentiation of MSCs by cDNA microarray. Table 2. Gene expression profiles during adipogenic differentiation of MSCs by cDNA microarray. Supplementary material related to this article can be found in the online version. [file 8069930.f1.docx]

**Supplementary Material**

**Icariin and Icariside Ⅱ Reciprocally Stimulate Osteogenesis and Inhibit Adipogenesis of Multipotential Stromal Cells through ERK Signaling**


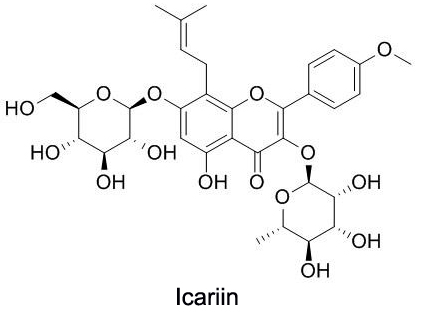

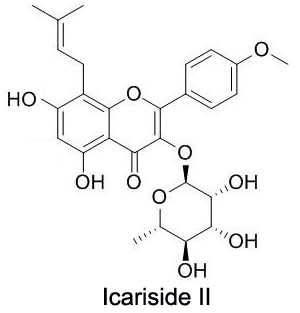


FIGURE 1: Chemical structure of icariin and icariside Ⅱ.

TABLE 1: Gene expression profiles during osteogenic **differentiation** of MSCs by cDNA microarray

| **Gene symbol** | **Full name** | **Fold changes** | |
| --- | --- | --- | --- |
|  |  | **OS** | **OS+ICA** |
| *Mt2* | Metallothionein2 | 2.19 | 2.28 |
| *Lrp1* | Low density lipoprotein receptor-related protein 1 | 2.17 | ^#^ |
| *Anp32b* | Acidic (leucine-rich) nuclear phosphoprotein 32 family, member B | 2.09 | ^#^ |
| *Nfkbia* | Nuclear factor of kappa light polypeptide gene enhancer in B cells inhibitor, alpha | 2.07 | ^#^ |
| *Srebf1* | Sterol regulatory element binding transcription factor 1 | 2.06 | 2.20 |
| *Mdm2* | Transformed mouse 3T3 cell double minute 2 | 2.05 | ^#^ |
| *Psmc6* | **Proteasome (prosome, macropain) 26S subunit, ATPase, 6** | 2.02 | ^#^ |
| *Srf* | Serum response factor (c-fos serum response element-binding transcription factor) | 0.49 | ^#^ |
| *E2f6* | E2F transcription factor 6 | 0.49 | ^#^ |
| *Prl2c2* | Prolactin family 2, subfamily c, member 2 | 0.49 | ^#^ |
| *Abcd4* | ATP-binding cassette, sub-family D (ALD), member 4 | 0.49 | ^#^ |
| *Smpd2* | Sphingomyelin phosphodiesterase 2, neutral | 0.47 | ^#^ |
| *Ptges3* | Prostaglandin E synthase 3 (cytosolic) | 0.47 | ^#^ |
| *Nedd8* | Neural precursor cell expressed, developmentally down-regulated gene 8 | 0.47 | ^#^ |
| *Pgk1* | Phosphoglycerate kinase 1 | 0.46 | ^#^ |
| *Nox1* | NADPH oxidase 1 | 0.46 | ^#^ |
| *Btk* | Brutonagammaglobulinemia tyrosine kinase | 0.46 | ^#^ |
| *Spin1* | Spindlin 1 | 0.46 | ^#^ |
| *Marcks* | Myristoylated alanine rich protein kinase C substrate | 0.45 | 0.49 |
| *Csf3r* | Colony stimulating factor 3 receptor (granulocyte) | 0.45 | ^#^ |
| *Naip1* | NLR family, apoptosis inhibitory protein 1 | 0.45 | ^#^ |
| *Igfbp3* | Insulin-like growth factor binding protein 3 | 0.45 | ^#^ |
| *Vdr* | Vitamin D receptor | 0.44 | ^#^ |
| *Ndufa13* | NADH dehydrogenase (ubiquinone) 1 alpha subcomplex, 13 | 0.43 | ^#^ |
| *Gfra1* | Glial cell line derived neurotrophic factor family receptor alpha 1 | 0.43 | ^#^ |
| *Ero1l* | ERO1-like (S. cerevisiae) | 0.41 | ^#^ |
| *Myl9* | Myosin, light polypeptide 9, regulatory | 0.41 | ^#^ |

Genes showed a greater than 2-fold induction or repression (Cy5/Cy3 ratios > 2 or < 0.5) were presented in TABLE 1. ^#^ 0.5 ≤ fold change ≤ 2. ICA, icariin.

TABLE 2: Gene expression profiles during adipogenic **differentiation** of MSCs by cDNA microarray

| **Gene symbol** | **Full name** | **Fold changes** | |
| --- | --- | --- | --- |
|  |  | **AS** | **AS+ICA** |
| *Ecm1* | Extracellular matrix protein 1 | 3.83 | 2.27 |
| *Gas1* | Growth arrest specific 1 | 3.79 | ^#^ |
| *Cbr2* | Carbonyl reductase 2 | 3.43 | 3.82 |
| *Fasn* | Fatty acid synthase | 3.05 | 2.08 |
| *Psmb5* | Proteasome (prosome, macropain) subunit, beta type 5 | 3.0 | ^#^ |
| *Mt2* | Metallothionein 2 | 2.59 | 3.46 |
| *C1qa* | Complement component 1, q subcomponent, alpha polypeptide | 2.35 | ^#^ |
| *Ghr* | Growth hormone receptor | 2.29 | ^#^ |
| *Ap2a2* | Adaptor-related protein complex 2, alpha 2 subunit | 2.27 | ^#^ |
| *Zfp36l1* | Zinc finger protein 36, C3H type-like 1 | 2.26 | ^#^ |
| *Etv5* | Ets variant 5 | 2.19 | ^#^ |
| *C1qc* | Complement component 1, q subcomponent, C chain | 2.15 | 2.08 |
| *Nfkbia* | Nuclear factor of kappa light polypeptide gene enhancer in B cells inhibitor, alpha | 2.07 | ^#^ |
| *C1qb* | Complement component 1, q subcomponent, beta polypeptide | 2.05 | 2.16 |
| *Wwp2* | WW domain containing E3 ubiquitin protein ligase 2 | 0.49 | 0.37 |
| *Serpine2* | Serine (or cysteine) peptidase inhibitor, clade E, member 2 | 0.48 | ^#^ |
| *Nfatc4* | Nuclear factor of activated T cells, cytoplasmic, calcineurin dependent 4 | 0.48 | 0.34 |
| *Ccna2* | Cyclin A2 | 0.47 | 2.04 |
| *E2f5* | E2F transcription factor 5 | 0.47 | ^#^ |
| *Marcks* | Myristoylated alanine rich protein kinase C substrate | 0.47 | 0.38 |
| *Hsp90aa1* | Heat shock protein 90, alpha (cytosolic), class A member 1 | 0.46 | 0.39 |
| *Creg1* | Cellular repressor of E1A-stimulated genes 1 | 0.45 | 0.49 |
| *Fermt2* | Fermitin family homolog 2 (Drosophila) | 0.45 | ^#^ |
| *Esyt1* | Extended synaptotagmin-like protein 1 | 0.44 | ^#^ |
| *Nfe2l2* | Nuclear factor, erythroid derived 2, like 2 | 0.43 | ^#^ |
| *Lpl* | Lipoprotein lipase | 0.42 | 0.36 |
| *Serpine1* | Serine (or cysteine) peptidase inhibitor, clade E, member 1 | 0.41 | 0.39 |
| *Ctsl* | Cathepsin L | 0.38 | 0.42 |
| *Psma6* | Proteasome (prosome, macropain) subunit, alpha type 6 | ^#^ | 3.07 |
| *Cltc* | Clathrin, heavy polypeptide (Hc) | ^#^ | 0.05 |

Genes showed a greater than 2-fold induction or repression (Cy5/Cy3 ratios > 2 or < 0.5) were presented in TABLE 2. ^#^ 0.5 ≤ fold change ≤ 2. ICA, icariin.
